# Evaluation of *Attalea tessmannii* Kernel Oil as a Feedstock for Biodiesel Production by Methanolysis and Ethanolysis

**DOI:** 10.1021/acsomega.6c03123

**Published:** 2026-10-15

**Authors:** Maurício Z. F. Arlindo, Emanuelle Q. Jardim, Luiz Henrique Han, Luiz Carlos dos Santos, Andrei V. Igansi, Henara L. Costa, Anderson F. Porte, Rafael Lipinski Paes, Débora P. Jaeschke, Luiz A. A. Pinto, Tito R. S. Cadaval Junior, Nauro da Silveira Junior

**Affiliations:** † Industrial Technology Laboratory, 67820 Federal University of Rio Grande − FURG, School of Chemistry and Food, Itália Avenue, km 8, unnumbered, 96203-900 Carreiros. Rio Grande, Rio Grande do Sul, Brazil; ‡ School of Engineering, Federal University of Rio Grande − FURG, School of Chemistry and Food − EQA. Itália Avenue, km 8, unnumbered, 96203-900 Carreiros. Rio Grande, Rio Grande do Sul, Brazil; § Federal Institute of Education, Science and Technology of Rio Grande do Sul (IFRS). 475 Eng. Alfredo Huck Street. Postal Code 96201-460, Downtown. Rio Grande, Rio Grande do Sul Brazil; ∥ Production Engineer Laboratory, School of Engineering, Federal University of Rio Grande − FURG., Itália Avenue, km 8, unnumbered, 96203-900 Carreiros. Rio Grande, Rio Grande do Sul, Brazil

## Abstract

Biodiesel production has been investigated due to its potential to mitigate pollutant gas emissions through the partial replacement of petroleum diesel. Although the technology is well-established, continued research focuses on the evaluation of alternative feedstocks and production routes that improve performance and economic viability. In this study, oil extracted from the kernels of *Attalea tessmannii*, a highly saturated oil obtained from an Amazonian fruit, was assessed as a feedstock for biodiesel production via methanolysis and ethanolysis. Crude and neutralized oils were subjected to base-catalyzed transesterification, and the resulting biofuels were characterized in terms of ester content, density, kinematic viscosity, acidity index, thermal behavior, and flash point. Oil refining resulted in an overall yield of 87%, while transesterification achieved ester contents of up to 93% for methanolysis and 97% for ethanolysis. The methanolysis products did not meet the minimum ester-content requirement specified by EN 14214 for FAME and also exhibited kinematic viscosities below the EN 14214 range. In contrast, the product obtained by ethanolysis of neutralized oil showed the most favorable overall fuel-quality profile among the evaluated routes, with density of approximately 863 kg m^–3^, kinematic viscosity near 3.1 mm^2^ s^–1^, acidity index below 0.5 mg KOH g^–1^, and flash point of 139 °C. A preliminary economic assessment revealed raw-material costs ranging from 0.536 to 0.712 USD kg^–1^. Although the crude oil–ethanol route presented the lowest production cost and the highest contribution margin per hour, the product obtained from this route exceeded the maximum acid-value limit considered in this study. These findings provide the first experimental evidence that *Attalea tessmannii* kernel oil is technically compatible with conventional base-catalyzed transesterification, with ethanolysis of neutralized oil offering the most favorable route among those evaluated.

## Graphical abstract

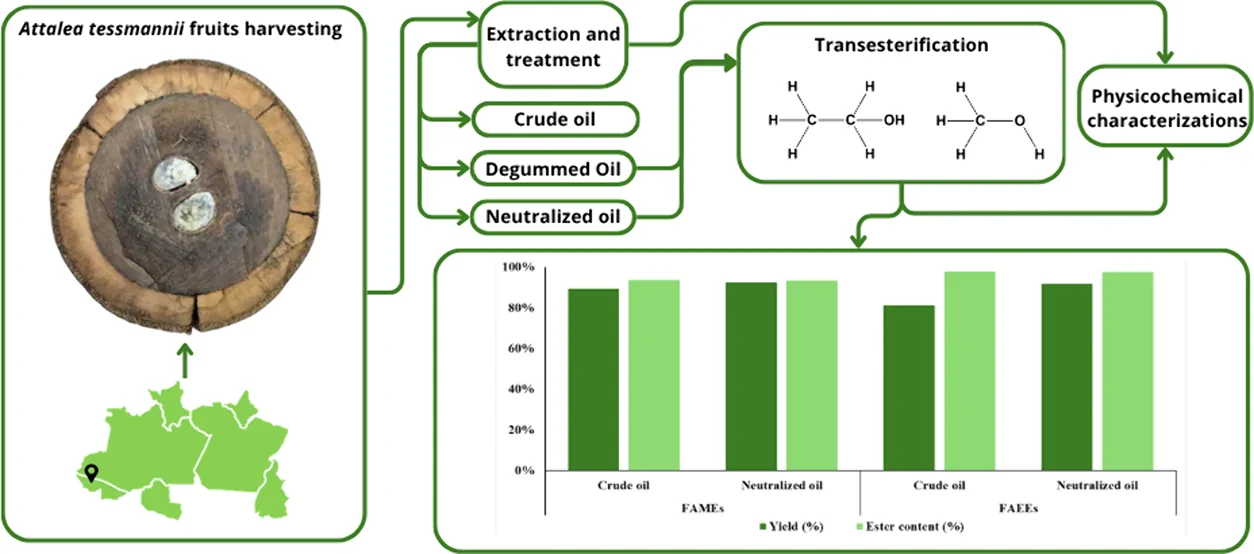

## 1 Introduction

The extensive use of fossil fuels over recent decades has demonstrated the urgent need for environmentally friendly alternatives, driven by their environmental and health impacts. Consequently, biobased and renewable materials have gained prominence for both sustainable energy production and environmental remediation purposes.^1–3^ Diesel is a widely used fuel, particularly due to properties that favor its application in the logistics and transportation sectors, including ships, buses, locomotives, generators, and other heavy machinery.^4^ Although diesel exhibits excellent combustion properties and is employed in long-lasting engines, it remains a fossil fuel, and its use is directly associated with significant emissions of atmospheric pollutants.^5^ Among the fuels capable of replacing diesel is biodiesel. In contrast to petroleum diesel, which consists of long-chain hydrocarbons, biodiesel is composed of fatty acid esters: fatty acid methyl esters (FAMEs) when methanol is used, and fatty acid ethyl esters (FAEEs) when ethanol is employed. Regarding emissions, biodiesel can reduce greenhouse gas emissions by up to 86% compared to conventional diesel, depending on the feedstock and production pathway.^6^ This reduction is attributed to the biogenic carbon cycle, in which the CO_2_ emitted during combustion is counterbalanced by its uptake during the growth of raw materials such as soybean and palm.

Although biodiesel has great potential for application and its use is widely encouraged, several challenges remain concerning both its use and production.^7^ Regarding engine performance, uncertainties remain about its compatibility with engine materials, as well as its physicochemical differences relative to diesel, including higher viscosity, lower oxidative stability, and susceptibility to cold-flow issues. These properties vary considerably and depend largely on the type of oil used as feedstock.^8,9^ However, the alcohol used during production also affects some biodiesel properties, with higher-chain alcohols generally improving cold-flow properties.^10–12^ As for oils, long-chain fatty acids yield biodiesel with higher viscosity and flash point, whereas shorter-chain oils have the opposite effect. Saturated oils generally lead to fuels with higher cetane numbers but exhibit higher solidification temperatures. In contrast, unsaturated oils reduce the cetane number, as it is proportional to ester chain length and inversely proportional to the degree of unsaturation.^13,14^ For cold-flow properties, unsaturated oils may result in lower solidification temperatures, allowing their use as feedstocks in colder climates.^15^ Thus, evaluating different feedstocks and the biodiesel produced from them is essential to support decentralized production tailored to regional conditions while ensuring high fuel quality. Regardless of the selected feedstock, however, appropriate pretreatment remains necessary. Unlike edible oils, oils intended for biodiesel production require degumming, neutralization, washing, and drying, but not deodorization or bleaching.^16^ Degumming removes phospholipids from crude oil, while neutralization eliminates free fatty acids that may form soaps during transesterification. Subsequent washing removes neutralization byproducts, and the oil is then dried to eliminate residual moisture.^17^

Biodiesel production methods rely on different types of catalysis: basic, acidic, or enzymatic. Basic catalysis, either homogeneous or heterogeneous, is the most employed for transesterification, using strong bases such as NaOH,^18,19^ or metal oxides such as CaO and MgO.^20,21^ However, soap formation due to reactions between free fatty acids and basic catalysts can significantly reduce yields. Acid catalysis is mainly applied to lower-quality oils, as acidic catalysts do not cause soap formation and promote efficient esterification of free fatty acids. Nevertheless, acid catalysis is less efficient for transesterification than basic catalysis and requires higher alcohol concentrations to achieve satisfactory conversions.^22,23^ Enzymatic catalysis employs triacylglycerol hydrolases (lipases) as catalysts. While enzymes provide high selectivity due to the specificity of their active sites, their reaction rates are slower compared to chemical catalysts.^24,25^ Enzymatic methods also avoid the formation of byproducts such as soaps and free fatty acids; however, enzymes require stricter control of temperature and pH to prevent denaturation and activity loss.

Despite the extensive use of conventional vegetable oils for biodiesel production, the search for alternative and regionally available feedstocks remains essential to reduce production costs, and enable decentralized biofuel production systems. In this context, the investigation of underexplored oleaginous materials with suitable fatty acid compositions is particularly relevant.^26^ A promising novel oil feedstock is the kernel of *Attalea tessmannii*, a nontimber forest palm species traditionally used by local communities in the Amazon region, where its fruits (“cocão”) are consumed as food and the kernel oil is used in regional culinary practices, typically obtained through small-scale cold pressing. The residual press cake is commonly reused for charcoal or fuel production, reflecting an integrated and circular use of the biomass at the community level.^27^ These almonds contain approximately 65% lipids by mass, the majority of which are nonpolar. According to Ahmad et. al,^27^ the fatty acid profile of the extracted oil also exhibits characteristics relevant for biodiesel production. Overall, the oil shows a high content of saturated fatty acids, around 90%, with roughly 50% consisting of lauric acid. The presence of short, highly saturated fatty acid chains suggests potential applicability of this oil as a biodiesel feedstock.

Another important factor is the geographical origin of this feedstock. *Attalea tessmannii* trees are native to the Amazon rainforest, and oil extracted from their kernels could support decentralized biodiesel production strategies in Amazonian regions, potentially reducing transportation costs. However, quantitative data on species abundance, fruit productivity, seasonal availability, and sustainable harvesting capacity remains limited, so this regional potential remains a hypothesis to be confirmed. Further agronomic, ecological, and socio-economic studies are still necessary to evaluate the long-term feasibility of large-scale biodiesel production based on this species.

While the fatty acid composition of *Attalea tessmannii* oil has been previously reported, studies addressing its refining behavior, transesterification performance, fuel-quality properties, and preliminary economic viability for biodiesel production remain unavailable. In addition, the highly lauric and saturated profile of this Amazonian oil provides an opportunity to investigate how feedstock composition influences biodiesel physicochemical behavior. Therefore, the present study aims to carry out the synthesis and characterization of biodiesel derived from *Attalea tessmannii* kernel oil at different refining steps, using a conventional base-catalyzed transesterification process. In addition, physicochemical characterizations or properties estimations of both the oil and the resulting biodiesel were performed to assess the suitability of this feedstock and to compare the experimentally determined fuel properties with relevant biodiesel specification limits. A preliminary economic assessment was also conducted, considering different stages of the oil refining process.

## 2 Experimental Section

### 2.1 Materials

Fruits of *Attalea tessmannii* were collected from their natural habitat in the Mogno State Forest, located in Tarauacá, Acre, Brazil (8°08′08.0″ S, 70°45′54.0″ W). Prior to oil extraction, the fruits were cut longitudinally using a saw to facilitate the removal of the kernels. The oil contained within the kernels was subsequently extracted by mechanical pressing, yielding the crude kernel oil for further analysis and processing. For the oil refining process, phosphoric acid (85%, w/w, Neon) and sodium hydroxide (P.A. Dinâmica) were used. In the transesterification reactions, methanol (P.A., Dinâmica), ethanol (99.5% v/v, Dinâmica) and sodium methoxide (30% in methanol, Sigma-Aldrich) were employed. For the chemical characterization, potassium hydroxide (P.A., Dinâmica), sulfuric acid (98% w/v, Dinâmica), HPLC grade methanol (Dinâmica), HPLC grade hexane (Dinâmica) and deuterated chloroform (Sigma-Aldrich) were employed.

### 2.2 Oil Treatment

The crude oil was treated according to Moraes et al.^17^ First, the oil was physically degummed. For that, it was heated to 80 °C, mixed with 3% w/w distilled water for 30 min at 300 rpm to remove phospholipids, and centrifuged (7, 000 g, 20 min). Then, the oil was reheated to 80 °C, treated with 1% w/w phosphoric acid for 30 min under 300 rpm, and centrifuged (7, 000 g, 20 min). The degummed oil was then neutralized using a 20% w/w NaOH solution, applied with a 4% excess relative to the acid value determined by NaOH titration. The phases were separated via centrifugation (7,000 g, 20 min). Residual NaOH was washed with distilled water at 40 °C (10% w/w) for 5 min, followed by a final centrifugation step (7,000 g, 20 min). Residual water was eliminated by rotary vacuum evaporation for 30 min at 110 °C. In addition, samples of both crude and physically degummed oil were dried by rotary vacuum evaporation prior to transesterification process and properties evaluation.

### 2.3 Transesterification and Biodiesel Purification

The transesterification process was conducted with crude and neutralized oil, employing reactions with methanol and ethanol. Physically degummed oil was not employed due to the high acidity index observed. Reactions were conducted in duplicates, and the conditions were selected based on commonly reported parameters for homogeneous alkali-catalyzed transesterification of vegetable oils.^28,29^ The alcohol-to-oil molar ratio, reaction temperature, reaction time, and agitation rate were chosen within the ranges commonly reported to promote efficient transesterification and minimize mass-transfer limitations. For reactions using methanol, the temperature was kept at 60 °C, whereas for ethanol reactions, it was set to 70 °C. For all reactions, sodium methoxide concentration was 1% w/w relative to the oil, and the alcohol-to-oil molar ratio was 6:1. All reactions were carried out for 60 min under agitation at 300 rpm.

After every transesterification process, the glycerol and biodiesel phases (lower and upper layers, respectively) were separated using a separatory funnel. The biodiesel was then washed with distilled water at 70 °C (3:1) to remove the remaining alcohol, catalyst, and soaps. The washing process was conducted until the biodiesel was clear and transparent. Afterward, biodiesel was dried out using rotatory vacuum evaporation (110 °C, 30 min).

### 2.4 Oil and Biodiesel Analysis

The fatty acid profiles of crude, degummed, and neutralized oil samples were determined using gas chromatography-flame ionization detector (GC-FID; Shimadzu 2010 Plus). For derivatization, 30 mg of oil sample was treated with 500 μL of a solution of KOH in HPLC-grade methanol. The mixture was homogenized in an ultrasonic bath and heated at 60 °C for 90 min in a water bath. Subsequently, 1.5 mL of a 1 M H_2_ SO_4_ solution was added, and the mixture was maintained at 60 °C for an additional 90 min. HPLC-grade hexane was then added for extraction. After phase separation, the hexane layer was collected for GC-FID analysis, following a procedure adapted from Hartman and Lago.^30^

Proton nuclear magnetic resonance (^1^H NMR, Bruker Ascend 400mhz) was employed to determine saponification value (SV) and iodine values (IV) in oil samples, following Santos et al.^31^ procedure. For biodiesel, ^1^H NMR analysis was used to determine the ester content in the samples for both methanolysis and ethanolysis products.^32^ All NMR samples were prepared using 50 μL of sample diluted in 550 μL of CDCl_3_, employing 32 scans, D1 equal to 7 and P1 equal to 14.35.

Differential scanning calorimetry (DSC) analysis was conducted for oil and biodiesel samples at a heating rate of 10 °C/min from −70 to 60 °C. The flashpoint temperature was evaluated for neutralized biodiesel samples following ASTM D93.^33^ The density was determined using an electronic densimeter (Anton Paar DMA-38), while the viscosity was obtained using ASTM D445 ^34^ method. The acidity index (AI) was determined for all samples and was carried out according to the Ca 5a-40 method.^35^ Cold flow properties and cetane number were estimated using literature models. Cetane number was estimated following the model proposed by Krisnangkura,^36^ using iodine and saponification values. Cold-flow properties were estimated by Gomes-Rodriguez et al.^10^ models, obtaining estimatives for cold filter plugging point (CFPP), cloud point (CP), and pour point (PP). Neutralized oil fatty profile was used in all calculations.

### 2.5 Preliminary Economic Analyses

A preliminary economic screening based on raw-material consumption and process yields was performed to compare four laboratory-scale processing scenarios combining different oil pretreatment levels (crude, and neutralized) with the use of methanol or ethanol as alcohol reagents: scenario 1 (crude oil – methanol); scenario 2 (crude oil – ethanol); scenario 3 (neutralized oil – methanol); scenario 4 (neutralized oil – ethanol). The economic assessment followed the process-cost estimation framework proposed by Douglas,^37^ considering the mass of raw materials and reagents required during the extraction, refining, and transesterification stages. The process mass balance was established from the experimental yields obtained in each unit operation, following the general procedures described in previous studies.^38,39^

The mass-balance procedure comprised the following steps: (i) development of the process flow diagram; (ii) identification of input and output streams (Figure 1), physical constraints, stoichiometric relationships, and known and unknown variables; (iii) degrees-of-freedom analysis; (iv) definition and solution of the governing mass-balance equations; (v) determination of the raw-material requirements for biodiesel production; and (vi) verification of overall mass-balance consistency.

**Figure 1:**
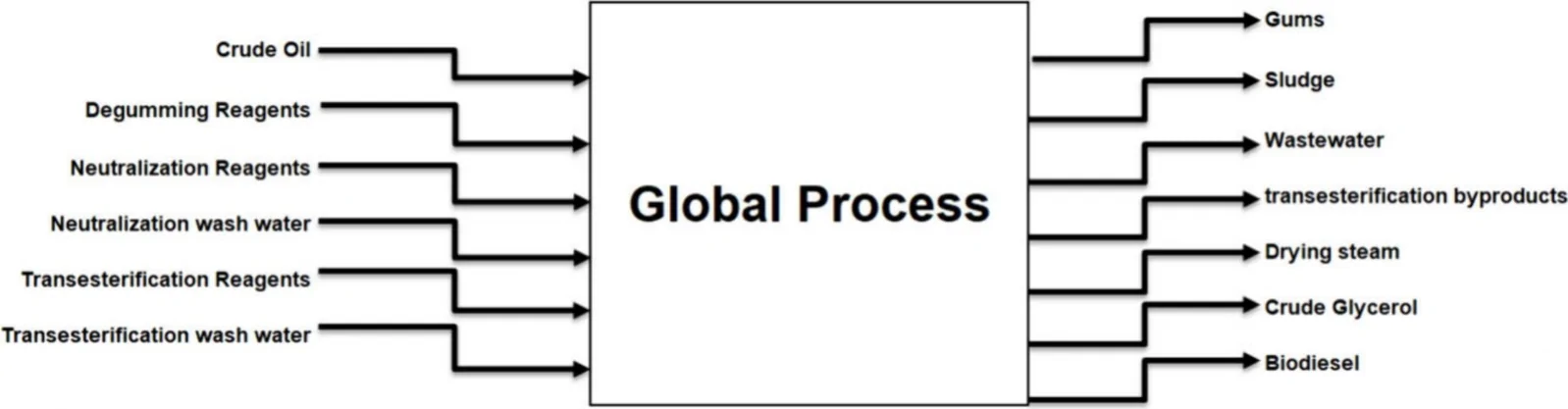
Input and output streams.

All economic calculations were normalized to an oil feed rate of 100 kg h^–1^, providing a common basis for comparison among the four scenarios.

1 $$C_{RM,h}=\Sigma \left(m_{i}\times P_{i}\right)$$

where *C*_*RM,h*_ is the total raw-material cost (USD h^–1^), *m*_*i*_ is the hourly consumption rate of raw material *i* (kg h^–1^), and *P*_*i*_ is the corresponding unit price (USD kg^–1^).

2 $$C_{RM,BD}=C_{RM,h}/m_{BD}$$

where *C*_*RM,BD*_ is the raw-material cost per kilogram of biodiesel (USD kg^–1^) and *m*_*BD*_ is the biodiesel production rate (kg h^–1^).

3 $$R_{h}=R_{BD,h}+R_{GLY,h}$$

Hourly revenue was calculated as the sum of the revenues generated by biodiesel and crude glycerol.

4 $$R_{BD,h}=m_{BD}\times P_{BD}$$

5 $$R_{GLY,h}=m_{GLY}\times P_{GLY}$$

where *R*_*BD,h*_ and *R*_*GLY,h*_ are the hourly revenues from biodiesel and crude glycerol (USD h^–1^), respectively; *m*_*BD*_ and *m*_*GLY*_ are the corresponding production rates (kg h^–1^); and *P*_*BD*_ and *P*_*GLY*_ are the adopted selling prices (USD kg^–1^).

6 $$CM_{h}=R_{h}-C_{RM,h}$$

where *CM*_*h*_ is the contribution margin (USD h^–1^), *R*_*h*_ is the total hourly revenue (USD h^–1^), and *C*_*RM,h*_ is the total hourly raw-material cost (USD h^–1^).

The reference prices adopted in the calculations were 0.20 USD kg^–1^ for crude oil, 5.00 USD kg^–1^ for H_3_PO_4_, 2.95 USD kg^–1^ for NaOH, approximately 0.0042 USD kg^–1^ for water, 8.04 USD kg^–1^ for sodium methoxide, 1.58 USD kg^–1^ for methanol, and 0.58 USD kg^–1^ for ethanol. The selling prices adopted for crude glycerol and biodiesel were 4.50 and 1.37 USD kg^–1^, respectively. Reference prices for process inputs and products were obtained from commercial market quotations, supplier information, and specialized market-price platforms available at the time of the assessment.

The present assessment represents a preliminary economic screening rather than a complete techno-economic analysis. Only raw material expenditures were considered as variable costs. Energy consumption, utilities, alcohol recovery, downstream separation, labor, maintenance, wastewater treatment, storage, logistics, depreciation, taxes, financing costs, and capital investment were outside the scope of the analysis. Accordingly, *CM*_*h*_ should be interpreted as a comparative gross contribution-margin indicator.

### 2.6 Statistical Analysis

All experiments and analytical measurements were performed, at least, in duplicate, and results are presented as mean ± standard deviation. Data were evaluated by analysis of variance (ANOVA) and Tukey test (95% confidence) using Statistica 13.5 (TIBCO Software Inc.).

## 3 Results and Discussion

### 3.1 Oil Refining

Figure 2 presents the oil yield obtained after each step, as well as the global yield of the treatment. A small loss of oil mass occurred after each refining step. During degumming, phospholipids were removed from the oil, whereas in the neutralization stage, the phosphoric acid added previously was consumed and free fatty acids were eliminated through soap formation.

**Figure 2:**
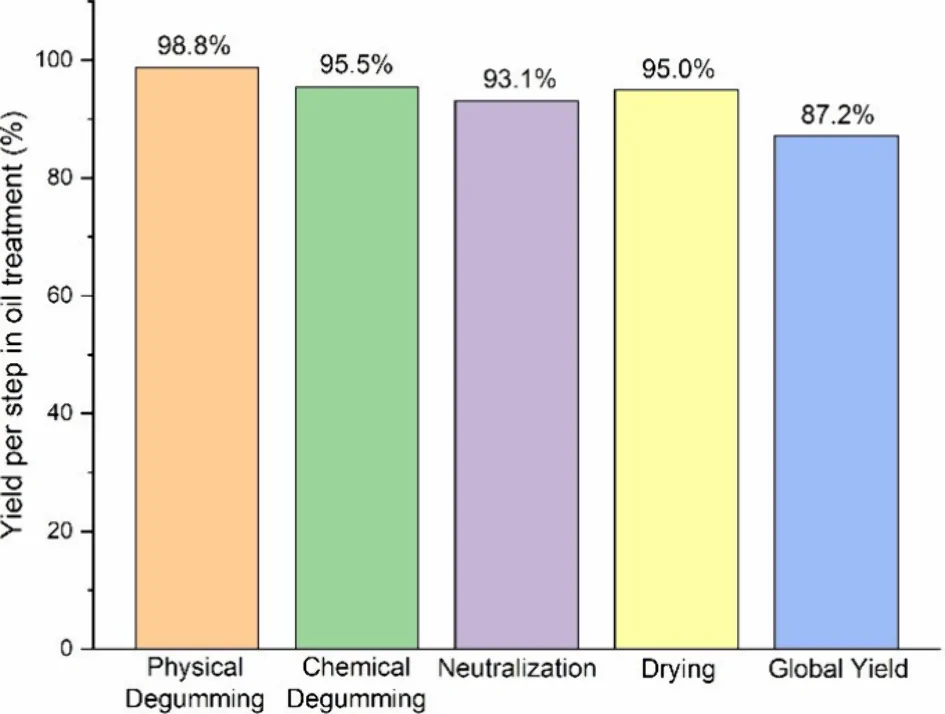
Yield obtained at each step of *Attalea tessmannii* oil refining process.

During the process, a clear reduction in oil turbidity was observed. Although turbidity in oils is often associated with the presence of water, it persisted even after drying the crude oil, indicating that moisture was not the cause. Instead, the turbidity was likely due to contaminants introduced during the pressing extraction process.

### 3.2 Oil Characterization

Table 1 presents the fatty acid methyl ester profile of the crude, physically degummed, and neutralized oil. Crude oil presented a high content of saturated fatty acids, composed mainly of lauric acid (dodecanoic acid). This profile is similar to the fatty profile reported by Ahmad et al.^27^ for *Attalea tessmannii* oil, with the main difference being the absence of trace amounts of certain fatty acids, such as caproic, arachidic, eicosenoic, heneicosanoic, behenic, and lignoceric acids (C6:0, C20:0, C20:1, C21:0, C22:0 and C24:0, respectively). This difference may be attributed to the extraction method used. In the present study, the oil was obtained by mechanical pressing, whereas Ahmad et al.^27^ extracted the oil using hexane, a solvent that recovers more nonpolar compounds.

**Table 1:** Fatty Acid Methyl Ester Profile for *Attalea tessmannii* Oil after Each Refining Step^a^

Fatty chain	Crude oil (area, %)	Physically degummed oil (area, %)	Neutralized oil (area, %)
C8:0	8.79 ± 0.85^a^	7.96 ± 0.08^a^	8 ± 0.14^a^
C10:0	7.8 ± 0.63^a^	7.15 ± 0.09^a^	7.05 ± 0.21^a^
C12:0	50.48 ± 0.27^a^	50.59 ± 0.33^a^	50.94 ± 0.71^a^
C14:0	14.37 ± 0.28^a^	14.33 ± 0.08^a^	14.09 ± 0.09^a^
C16:0	6.13 ± 0.99^a^	6.94 ± 0.06^a^	6.67 ± 0.07^a^
C18:0	2.48 ± 0.39^a^	2.88 ± 0.02^a^	3.14 ± 0.4^a^
C18:1	8.29 ± 0.09^a^	8.4 ± 0.03^a^	8.45 ± 0.31^a^
C18:2	1.67 ± 0.01^a^	1.75 ± 0.03^a^	1.66 ± 0.09^a^

a Means with equal letters in the same line do not present a significant difference (*p* > 0.05).

Compared with conventional biodiesel feedstocks, *Attalea tessmannii* oil exhibits a markedly more saturated fatty acid profile. Soybean oil, one of the most widely used biodiesel feedstocks, is characterized by high linoleic acid contents (typically 48–59%) and relatively low concentrations of palmitic and stearic acids, approximately 10% and 3%, respectively. Palm oil, in contrast, contains approximately 40% oleic acid and 45% palmitic acid.^40^ Among lauric-rich oils, the fatty acid composition of *A. tessmannii* is more similar to those of babassu, coconut, murumuru, and tucumã almond oils, all of which contain elevated levels of lauric acid.^41,42^ Murumuru seed fat, another Amazonian lipid source, also presents high lauric acid contents, although with a greater proportion of palmitic acid. Due to this composition, *A. tessmannii* demonstrates physicochemical characteristics favorable for biodiesel production and fuel properties comparable to other saturated lauric oils. However, *A. tessmannii* is still obtained predominantly from wild or semimanaged populations, limiting its current availability and large-scale applicability.

This highly saturated profile favors the production of biodiesel with a high cetane number, as it results in a high cetane number, which increases with both chain length and degree of saturation.^14^ It is also noteworthy that the treatment steps employed did not alter the fatty profile, as only minor differences were observed among the samples. Based on this profile, the mean molecular weight of the oil was estimated to be around 600g mol^–1^.

Table 2 presents the AI, SV and IV measured for the oil samples. The acid index must be kept as low as possible in order to enable base-catalyzed transesterification, as free fatty acids can react with the basic catalyst to form soap, thereby reducing the overall process yield. The different superscript letters indicate significant differences in acidity index among treatments (*p* < 0.05). Neutralization effectively reduced the acidity of the oil, resulting in the lowest AI among the evaluated samples, whereas physical degumming slightly increased this parameter. The higher AI observed in the physically degummed oil is related to partial hydrolysis of triglycerides, which can occur when the oil is heated in the presence of water, generating free fatty acids. The crude oil presented an AI similar to babassu oil.^42^ The AI results suggest that the neutralized oil is more likely to achieve a higher ester content during transesterification.

**Table 2:** Acidity Index (AI), Saponification Value (SV), and Iodine Value (IV) of *Attalea tessmannii* Crude, Physically Degummed and Neutralized Oils

Sample	AI (mg KOH g^–1^)	SV (mg KOH g^–1^)	IV (cg I_2_ g^–1^)
Crude oil	0.895 ± 0.026^b^	255.40 ± 3.53^a^	12.58 ± 0.70^a^
Physically degummed oil	0.993 ± 0.008^a^	253.49 ± 5.62^a^	11.84 ± 1.15^a^
Neutralized oil	0.201 ± 0.011^c^	257.09 ± 2.58^a^	12.04 ± 1.05^a^

The SV of *Attalea tessmannii* oil (approximately 259 mg KOH g^–1^) is similar to that of coconut and babassu oil, which are reported to be around 249 mg KOH g^–1^.^42,43^ These values are higher than those typically reported for soybean oil (approximately 190 mg KOH g^–144^, due to the shorter fatty acid chain profile characteristic of *Attalea tessmannii* oil. Regarding the IV, the values obtained in the present work are consistent with previous findings.^27^ When compared with soybean oil, which typically presents an IV of 124–139 cg I_2_ g^–1^ due to its high content of unsaturated fatty acids,^40^ *Attalea tessmannii* oil showed a lower IV, reflecting its higher degree of saturation. This low IV is comparable to those of other highly saturated oils and fats, such as babassu oil (6–11 cg I_2_ g^–1^),^42,45^ coconut oil (13 to 17 cg I_2_ g^–1^)^46,47^ and murumuru fat (11 cg I_2_ g^–1^).^42^

Table 3 presents the density, viscosity, and phase transition properties of the crude, physically degummed, and neutralized oils. No significant differences were observed in viscosity among the oil samples (*p* > 0.05), while density values remained constant throughout the refining process. The absence of variation among replicates is related to the measurement resolution of the densimeter. These values are similar to those reported for babassu oil density.^45^ The results indicate that degumming and neutralization mainly affected the free fatty acid content of the oil (Table 2), with minimal impact on its physical properties. When compared with other vegetable oils, the kinematic viscosity values obtained were lower. Soybean, rapeseed, and sunflower oils exhibit kinematic viscosities of approximately 39 mm^2^/s,^44^ which can be attributed to the greater molar mass of these oils. In comparison with oils of similar fatty acid profiles, the viscosity values were comparable, with babassu oil presenting a kinematic viscosity of approximately 29 mm^2^/s.^45^ Thermal analysis showed a phase transition peak near to 21 °C for all samples, with melting enthalpy around 110 kJ kg^–1^, also compared to babassu oil.^45^ This relatively high melting temperature is related to the highly saturated fatty profile of the oil. Notably, at room temperature, solidified *Attalea tessmannii* oil did not melt without heating, and the liquefied oil required cooling to resolidify. Compared with other saturated lipid sources, such as murumuru and tucumã fats, the phase transition peaks of *Attalea tessmannii* oil were similar. Aragão et al.^48^ reported melting temperatures of approximately 25 °C for murumuru fat and 23 °C for Tucumã fat. The energy required for the phase transition was also very similar to that observed for murumuru fat. Cupuaçu fat, another lipid source from the Amazon rainforest, exhibits a highly saturated profile, although composed of slightly longer fatty acid chains compared with *Attalea tessmannii* oil, resulting in a melting temperature of approximately 26 °C.^49^

**Table 3:** Density, Viscosity, and Thermal Properties of *Attalea tessmannii* Oil

Sample	Density at 20 °C (kg m^–3^)	Kinematic Viscosity at 40 °C (mm^2^ s^–1^)	Phase transition peak (°C)	ΔH (kJ kg^–1^)
Crude oil	921 ± 0	29.38 ± 0.61^a^	21.99 ± 0.97^a^	–113.75 ± 1.84^a^
Physically degummed oil	920 ± 0	30.09 ± 0.23^a^	21.61 ± 0.82^a^	–110.48 ± 5.29^a^
Neutralized oil	920 ± 0	29.25 ± 0.14^a^	22.13 ± 0.65^a^	–117.64 ± 3.97^a^

### 3.3 Transesterification Reactions

Table 4 presents the yield obtained for each reaction after purification, along with the ester content of each sample. The reactions were conducted with crude and neutralized *Attalea tessmannii* oil, since a low gum content was observed and the degumming process raised the acidity index of the oil. Statistical analysis revealed significant differences in both biodiesel yield and ester content among the evaluated treatments (*p* < 0.05). Ethanolysis of crude oil resulted in the lowest yield, while neutralization eliminated the differences between methanolysis and ethanolysis yields. For ester content, ethanolysis produced significantly higher values than methanolysis regardless of feedstock pretreatment, whereas no significant differences were observed between crude and neutralized oils within the same alcohol route.

**Table 4:** Yields and Ester Contents Obtained for the Transesterification Reactions

	Yield (%)		Ester content (%)	
Feedstock oil	Methanolysis	Ethanolysis	Methanolysis	Ethanolysis
Crude oil	89.16 ± 1.95^a^	81.28 ± 4.33^b^	93.52 ± 2.71^b^	97.72 ± 0.24^a^
Neutralized oil	92.56 ± 1.62^a^	91.48 ± 2.16^a^	93.28 ± 0.55^b^	97.40 ± 0.12^a^

Although crude oil exhibits a significantly high acid value compared to the neutralized oil, it is important to evaluate the transesterification yields in order to elucidate whether the process can be economically viable when using crude oil as the feedstock. In general, transesterification reactions carried out with ethanol showed reduced mass yields due to soap formation, with the exception of those performed using neutralized oil. The reduction in biodiesel yield is a common consequence of base-catalyzed reactions, as the formation of soaps leads to product losses during the washing step.^50^ Methanolysis reactions presented much lower turbidity than ethanolysis reactions, which is attributed to the higher water content presented in ethanol. This residual water promotes saponification during transesterification, decreasing the final mass yield. Although soap formation reduces the yield, it does not directly affect the ester content of the final biodiesel.

Regarding the regulatory framework, the European Union standard (EN 14214)^51^ specifies a minimum ester content of 96.5% strictly for FAMEs, whereas ASTM D6751–24 ^52^ does not establish a minimum total ester content for biodiesel. Therefore, the 96.5% threshold specified by EN 14214 was applied directly to the FAME samples obtained by methanolysis. For the FAEE samples obtained by ethanolysis, this value was used solely as a comparative benchmark and not as a criterion for regulatory compliance. Based on this distinction, both ethanolysis products reached or exceeded the 96.5% benchmark, whereas the methanolysis products did not meet the minimum ester content specified by EN 14214 for FAME. The lower ester content observed in the methanolysis can likely be attributed to methanol volatilization, given that the reaction was carried out at 60 °C—a temperature near the alcohol’s boiling point.^51^ The alcohol-to-oil molar ratio of 6:1 is commonly employed to shift the transesterification equilibrium toward ester formation; however, partial methanol evaporation may reduce its effective concentration in the liquid phase, affecting reaction equilibrium and reducing ester formation. Literature reports indicate that methanol is generally more reactive than ethanol in transesterification reactions, leading to faster kinetics and higher ester yields under similar conditions.^53,54^ Therefore, the lower ester content observed in methanolysis suggests that methanol volatilization may have contributed to reducing transesterification efficiency. Nevertheless, this effect was not directly measured in the present study and should be considered only as a possible contributing factor rather than a definitive explanation.

Another factor that may have influenced the lower methyl ester content may be related to the quantification method based on ^1^H NMR analysis. Although this technique is widely used for estimating biodiesel conversion, some deviations compared to standardized analytical methods may occur. Braga et al.,^32^ for example, reported slightly lower conversion values determined by ^1^H NMR for biodiesel produced from safflower and babassu oils when compared to results obtained using the EN ISO standard method. In addition, the transesterification conditions employed in the present study were not specifically optimized for *Attalea tessmannii* oil but were selected based on reaction conditions commonly described in the literature for vegetable oil transesterification. Therefore, further optimization of parameters such as reaction temperature, alcohol-to-oil molar ratio, catalyst loading, and reaction time may improve ester conversion levels. Reaction parameters such as alcohol-to-oil molar ratio, reaction time, temperature, and agitation rate are known to strongly influence ester content and acidity. Higher alcohol excess generally favors ester formation by shifting the reaction equilibrium toward the products, while sufficient reaction time allows more complete triglyceride conversion.^55^ Temperature affects reaction kinetics and mass transfer, although excessive temperatures may promote alcohol losses.^56^ Agitation improves contact between the immiscible alcohol and oil phases, reducing mass-transfer limitations.^57^ Therefore, optimization of these parameters could potentially increase ester conversion and further reduce acidity in biodiesel produced from *Attalea tessmannii* oil.

### 3.4 Biodiesel Characterization

Table 5 presents the density, viscosity, and AI of the products obtained from crude and neutralized oils. Regarding physical properties, the European standard EN 14214 specifically applies to fatty acid methyl esters (FAME), establishing a kinematic viscosity range of 3.5–5.0 mm^2^ s^–1^ and a density range of 860–900 kg m^–3^. In contrast, the ASTM biodiesel specification encompasses monoalkyl esters of long-chain fatty acids and establishes a broader kinematic viscosity range of 1.9–6.0 mm^2^ s^–1^. Therefore, the EN 14214 limits were used as reference benchmarks for the FAEE samples rather than as a basis for claiming regulatory compliance. The ASTM and EN 14214 specification limits were used herein to compare the measured fuel properties of the synthesized products.

**Table 5:** Density, Viscosity, and Acidity Index (AI) of the Products Obtained from Crude and Neutralized Oils^a^

Feedstock oil	Density (kg m^–3^)	Kinematic viscosity (mm^2^ s^–1^)	AI (KOH mg g^–1^)
Crude oil + methanolysis	866 ± 0	2.81 ± 0.03^c^	0.354 ± 0.05^b,c^
Crude oil + ethanolysis	863 ± 0	3.16 ± 0.05^a^	0.736 ± 0.09^a^
Neutralized oil + methanolysis	867 ± 0	2.84 ± 0.02^c^	0.24 ± 0.01^c^
Neutralized oil + ethanolysis	863 ± 0	3.12 ± 0.02^b^	0.441 ± 0.01^b^

a Means followed by different lowercase letters within the same column differ significantly according to Tukey’s test (*p* < 0.05).

Statistical analysis revealed significant differences in both kinematic viscosity and acidity index among the biofuel samples (*p* < 0.05). Products obtained by ethanolysis exhibited significantly higher viscosity values than those obtained by methanolysis, while neutralization slightly reduced viscosity within the ethanolysis route. The methanolysis products exhibited kinematic viscosities below the range specified by EN 14214 for FAME, although their values were within the broader viscosity range established by ASTM D6751. The ethanolysis products showed viscosity values close to the lower EN 14214 limit used as a comparative benchmark for FAEE.

The density values showed less pronounced variation among the evaluated products. The FAEE products exhibited densities within the EN 14214 range used as a comparative benchmark, whereas the applicability of this range to FAEE should not be interpreted as evidence of regulatory compliance. The viscosity values are similar to those reported for babassu oil biodiesel (3.5 mm^2^ s^–1^),^58^ which can be attributed to the similar lipid profiles of these feedstocks.

Another particularly important property evaluated was the AI. As shown in Table 5, all samples obtained by methanolysis exhibited AI values below 0.5 KOH mg g^–1^, which is the maximum limit established by the aforementioned standards. In contrast, for ethanolysis, only the product obtained from neutralized oil presented AI below this limit. Therefore, despite the favorable economic performance observed for the crude oil–ethanol route, its acidity index remained above the maximum specification limit considered in this study. This result may be related to the presence of water in ethanol, which promotes not only soap formation but also oil hydrolysis and incomplete transesterification. This emphasizes the importance of oil neutralization for biodiesel production, since low AI is important for maintaining engine integrity. In addition to feedstock acidity and alcohol quality, process variables such as alcohol-to-oil molar ratio, reaction time, temperature, and mixing intensity are also known to influence acidity by affecting the extent of free fatty acid conversion during transesterification.^29^ Therefore, optimization of these parameters may contribute to further reductions in acidity values, particularly when processing feedstocks with higher initial free fatty acid contents.

Cetane number was estimated using the model described by Krisnangkura,^36^ which uses saponification and iodine values. Data from neutralized *Attalea tessmannii* oil was used in the estimative, which yielded a cetane value of 64.4. The value obtained is near the value described in literature for coconut oil^14,46^ and babassu oil,^59^ once both feedstocks present a similar fatty acid profile. Oils with a higher unsaturation degree, such as soybean and canola, present a cetane index below 50,^14^ being near or even inferior for ASTM and EN 14214.^51,52^

Considering the experimentally evaluated parameters, the product obtained from neutralized oil by ethanolysis showed the most favorable overall fuel-quality profile among the evaluated routes. Its measured properties fell within the corresponding specification limits or reference benchmarks considered in this study. In contrast, the methanolysis products did not meet the EN 14214 minimum ester-content requirement for FAME, while the product obtained from crude oil by ethanolysis exceeded the maximum acid-value limit considered.

### 3.5 Biodiesel Thermal Properties

DSC analyses were performed to evaluate the phase transition regions of the biodiesel samples and to compare them with the feedstock oil. Figure 3 shows the DSC thermogram of the different oil samples and their corresponding methyl and ethyl esters. The transesterification reaction converts triglycerides molecules into ester molecules, which lowers the boiling and solidification points of the resulting products. A significant reduction in the melting point occurs after the transesterification process. For the oil samples, similar melting point were observed, around 20 °C. This elevated melting point is attributed to the predominance of saturated fatty chains. The ester samples also exhibited similar profiles when considering the feedstock oil used; however, differences emerged depending on whether methanol or ethanol was employed.

**Figure 3:**
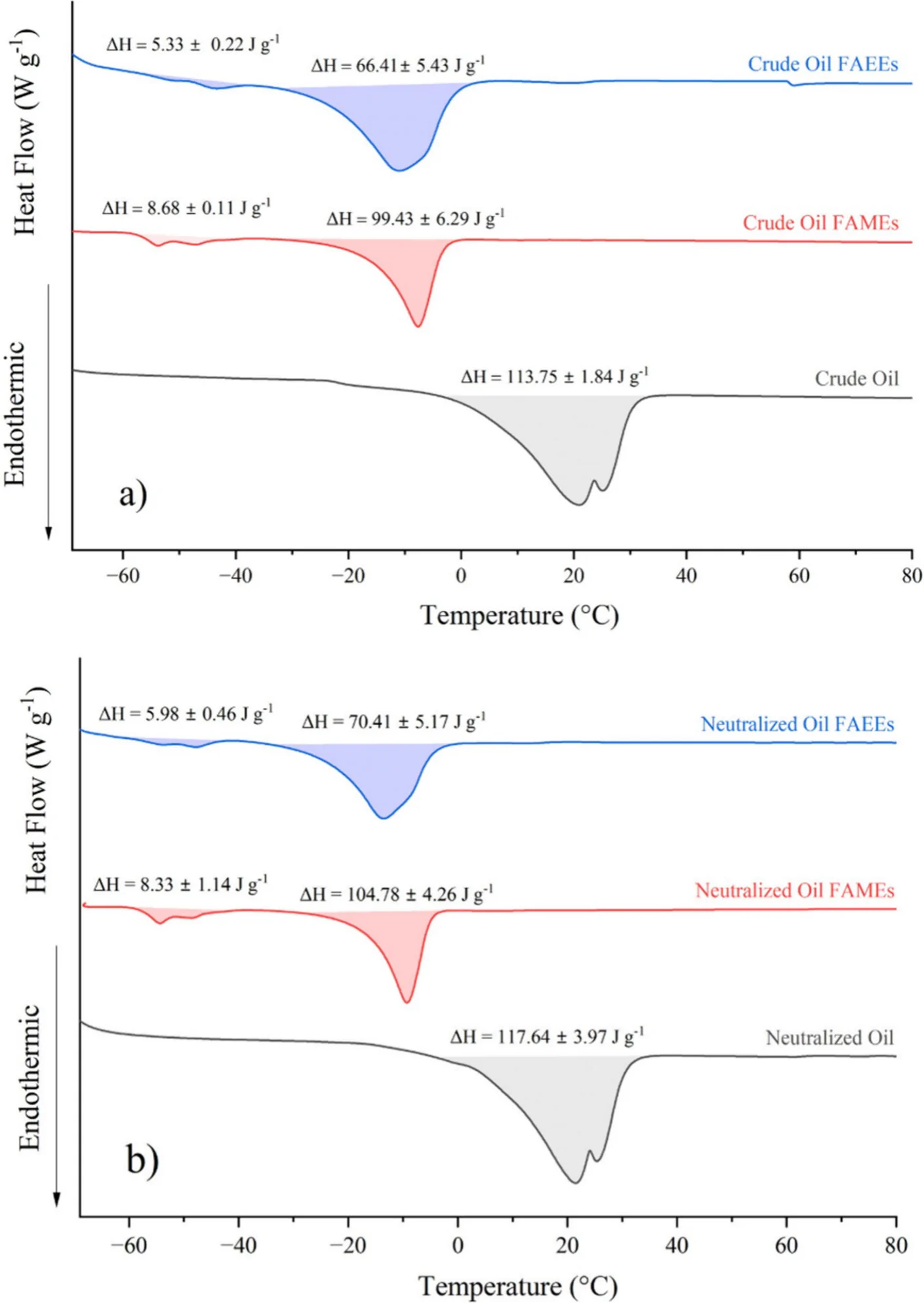
DSC thermograms of *Attalea tessmannii* (a) crude oil and its fuel samples, and (b) neutralized oil and its fuel samples.

The FAMEs samples showed a higher melting point compared to the FAEEs samples (−7.41 °C and −13.49 °C for crude oil FAMEs and FAEEs respectievely; −8.71 °C and −13.52 °C for neutralized oil FAMEs and FAEEs). This behavior is mainly associated with the longer alkyl chain inserted during ethanolysis. This extra methylenic group changes the capability of the liquid to form a solid structure while being cooled, which then leads to a lower melting point. This behavior is mainly associated with the different alkyl groups introduced during transesterification, since the temperature ranges of the phase transitions were very similar. Although the results were consistent among samples, the DSC analysis provided important information. In cold climates, biodiesel synthesized from *Attalea tessmannii* oil can solidify, which does not exclude its use but limits the region where it can be used.

Flash point was also determined for the esters produced from neutralized oil. The flashpoint of methyl esters was 106 ± 3.46 °C, whereas the ethyl esters exhibited a higher value of 139 ± 1.41 °C. Owing to its shorter fatty chain composition, *Attalea tessmannii* biodiesel presented a lower flashpoint when compared with soybean biodiesel (165 °C),^44^ but closer values when compared with biodiesel produced from babassu oil (115 °C).^58^ The ethyl ester derived from neutralized oil presented a higher flash point due to the longer side chain, resulting in a value above the minimum limits considered in this study. In contrast, the methyl biodiesel exhibited a flash point value below the minimum specified by ASTM standards for B100 (130 °C), although it remained above the minimum limit set by EU regulations.^51,52^

Cold-flow properties (CFPs) were estimated and are shown in Figure 4. The model used for this estimation is based on the fatty acid profile of FAMEs, and neutralized oil profile was considered for all calculations. The values obtained for CFPP, CP and PP for *Attalea tessmannii* FAMEs were similar to estimated and experimental values for coconut oil methyl esters,^14,46,47,60^ which has a very similar fatty acid profile. FAEEs (and other longer or branched esters) can exhibit slightly more favorable CFPs compared to FAMEs with the same fatty acid profile. The presence of an additional -*CH*_2_ unit at the carbonyl end of FAEE molecules decreases the overall amphiphilic nature of the structure, resulting in lower cloud points (CP) and melting points. Furthermore, a larger alkyl group (e.g., ethyl versus methyl) is sufficient to shield the interactions between the polar parts of the headgroup, disrupting the space between individual molecules in the crystal structure and yielding improved CFPs.^61^ It is important to note that the applied model was developed for fatty acid methyl esters (FAMEs) and has not been validated for FAEEs; therefore, the estimated values reported here refer only to the FAME products.

**Figure 4:**
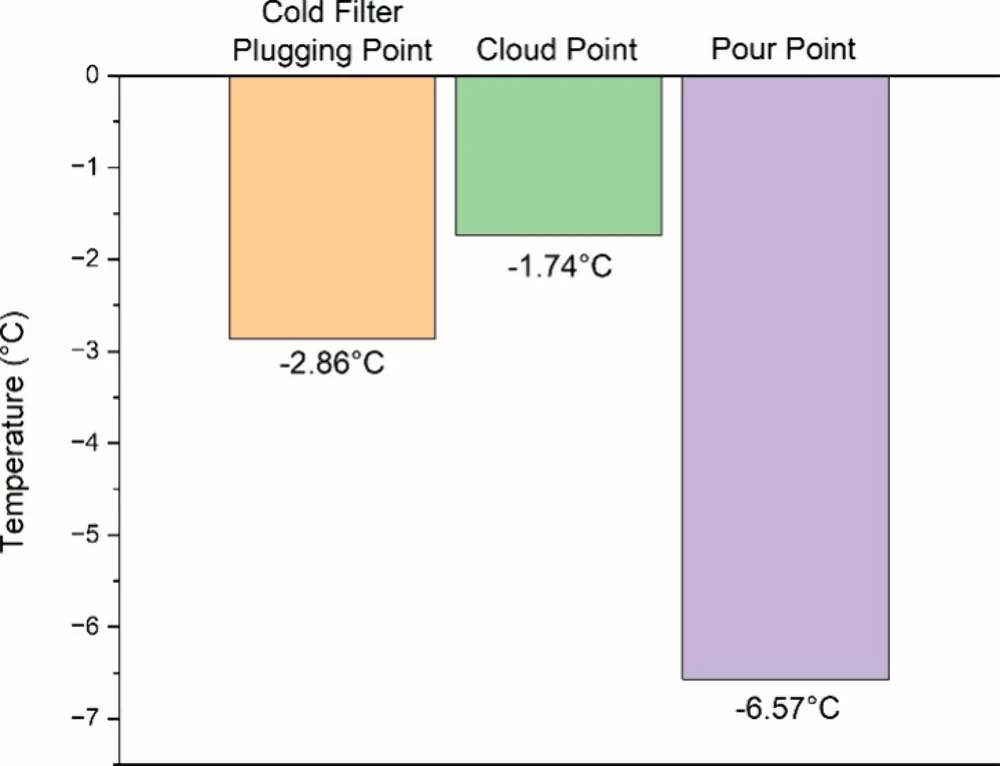
Estimated cold-flow properties of biodiesel obtained from *Attalea tessmannii* oil.

### 3.6 Preliminary Economic Assessment

The resulting biodiesel and crude-glycerol production rates, raw-material costs, total revenues, and contribution margins are summarized in Table 6.

**Table 6:** Consolidated Economic Indicators for the Four Processing Scenarios

Economic Indicator	Scenario 1 Crude Oil–Methanol	Scenario 2 Crude Oil–Ethanol	Scenario 3 Neutralized Oil–Methanol	Scenario 4 Neutralized Oil–Ethanol
Biodiesel production (kg h^–1^)	89.00	81.00	81.00	82.00
Crude-glycerol production (kg h^–1^)	14.35	14.01	12.40	12.09
Raw-material cost (USD h^–1^)	56.94	43.60	57.55	45.98
Raw-material cost (USD kg^–1^ biodiesel)	0.64	0.54	0.71	0.56
Total revenue (USD h^–1^)	186.71	174.39	166.63	166.53
Total revenue (USD kg^–1^ biodiesel)	2.09	2.15	2.06	2.03
Contribution margin, *CM*_*h*_ (USD h^–1^)	129.77	130.79	109.08	120.55

The results indicate a consistent economic advantage of ethanol over methanol under the assumptions adopted in this preliminary assessment. For both crude and neutralized oils, the ethanol-based routes resulted in lower raw-material expenditures than their corresponding methanol-based alternatives. Scenario 2 (crude oil–ethanol) presented the lowest raw-material cost, whereas Scenario 3 (neutralized oil–methanol) showed the highest. The use of ethanol reduced hourly raw-material expenditure by approximately 23% for crude oil and 20% for neutralized oil compared with the corresponding methanol-based routes.

Scenario 2 also presented the highest contribution margin, although its value was very close to that of Scenario 1. This result is noteworthy because Scenario 1 exhibited the highest biodiesel production rate and total hourly revenue. The lower raw-material expenditure of Scenario 2 compensated for its lower biodiesel production rate, resulting in a slightly higher contribution margin. Among the neutralized-oil routes, Scenario 4 showed a higher contribution margin than Scenario 3, further indicating the economic advantage of ethanol under the assumptions considered.

The economic ranking should, however, be considered together with the experimentally evaluated fuel-quality properties. Although Scenario 2 exhibited the most favorable raw-material economics, the product obtained from crude-oil ethanolysis exceeded the maximum acid-value limit considered in this study. In contrast, Scenario 4 showed more favorable results for the evaluated fuel-quality properties while retaining comparatively favorable economic performance. Thus, Scenario 2 was the most favorable route from the perspective of raw-material economics, whereas Scenario 4 provided a more favorable compromise between preliminary economic performance and the fuel-quality properties evaluated in this study.

These results should be interpreted within the scope of the preliminary assessment. The contribution margin considered revenues from biodiesel and crude glycerol and only raw-material expenditures as variable costs. Therefore, the reported values represent comparative indicators for the four laboratory-scale processing scenarios and should not be interpreted as net profit or commercial-scale process profitability.

## 4 Conclusion

This work investigates the biodiesel synthesis process using *Attalea tessmannii* oil, an underexplored Amazonian feedstock, providing to our knowledge the first experimental characterization of biodiesel derived from this species. Overall, the results demonstrate that this highly saturated, lauric-rich oil is technically compatible with base-catalyzed transesterification, with ethanolysis of neutralized oil providing the most favorable fuel-quality properties among the routes evaluated. The results demonstrated the technical feasibility of biodiesel production, with conversion levels of approximately 93% for methanolysis and 97% for ethanolysis. The observed fuel properties, including phase transition behavior (experimentally determined) and cetane number (estimated via empirical models), are consistent with the structure–property relationships previously established for other highly lauric, saturated oils such as coconut, babassu, and palm kernel, confirming that *Attalea tessmannii* oil behaves as expected for this class of feedstock and providing baseline empirical data to support future studies on this specific species. Furthermore, essential physicochemical properties of the synthesized fuels were evaluated. Regarding the EN 14214 specification, which specifically applies to FAME, the FAMEs synthesized from this Amazonian oil did not meet the evaluated requirements, particularly because of their low ester contents and kinematic viscosities below the specified range. Although process optimization may improve conversion, the methanolysis products did not meet the ester-content requirement under the conditions evaluated. FAEEs, on the other hand, presented more favorable results, with all measured properties of the product obtained from neutralized oil falling within the specification limits used as reference benchmarks in this study. These comparisons, particularly those with EN 14214, should not be interpreted as demonstrating formal regulatory compliance of the FAEE products. The FAEE produced from crude oil exceeded the maximum acidity value used as a reference in this study. Therefore, pretreatment steps remain important for improving the fuel-quality properties of biodiesel derived from *Attalea tessmannii* oil. Further experimental determination of CFPs and other standard properties (e.g., water content, free and total glycerin) is required. From a raw-material cost perspective, the crude oil routes generally showed higher contribution margins under the assumptions adopted in the preliminary assessment. However, additional studies including energy consumption, separation requirements, utilities, and capital expenditures are necessary to fully assess the industrial feasibility of the proposed biodiesel production routes. Considering both the evaluated fuel-quality properties and raw-material cost, no single route was superior across both criteria: the crude oil–ethanol route presented the lowest raw-material cost and highest contribution margin per hour but exceeded the acidity limit used as a reference, while the neutralized oil–ethanol route showed values within the evaluated reference limits at a higher raw-material cost.

The low iodine value of the oil indicates a low degree of unsaturation in its fatty acid composition, suggesting greater resistance to oxidative degradation and, consequently, a tendency to produce biodiesel with good oxidative stability, although this property was not experimentally evaluated in the present study. Nevertheless, the highly saturated fatty acid profile of *Attalea tessmannii* results in relatively limited cold-flow performance, which may limit the use of this biodiesel in colder regions without blending or other cold-flow improvement strategies. This study helps to understand the suitability of *Attalea tessmannii* oil in biodiesel synthesis, since this oil has a highly saturated profile and can be evaluated as a feedstock. Nevertheless, the regional relevance of *Attalea tessmannii* as a feedstock remains a hypothesis, and additional environmental, agronomic, and socio-economic studies are required to evaluate the sustainability and large-scale applicability of this feedstock in Amazonian regions. Future investigations addressing biomass availability, annual oil productivity, and sustainable harvesting capacity are necessary before the feasibility of *Attalea tessmannii* as a regional biodiesel feedstock can be evaluated.
